# Heart failure therapy in diabetic patients-comparison with the recent ESC/EASD guideline

**DOI:** 10.1186/1475-2840-10-15

**Published:** 2011-02-08

**Authors:** Frank Edelmann, Rolf Wachter, Hans-Dirk Düngen, Stefan Störk, Annette Richter, Raoul Stahrenberg, Till Neumann, Claus Lüers, Christiane E Angermann, Felix Mehrhof, Götz Gelbrich, Burkert Pieske

**Affiliations:** 1 Department of Cardiology and Pneumology, University of Göttingen, Göttingen, Germany; 2 Department of Cardiology, Campus Virchow, Charité University, Berlin, Germany; 3 Medical Clinic and Policlinic I, University of Würzburg, Würzburg, Germany; 4 Clinic for Internal Medicine and Cardiology, University of Marburg, Marburg, Germany; 5 Department of Cardiology, University Duisburg-Essen, Germany; 6 Clinical Trial Center, University of Leipzig, Leipzig, Germany; 7 Department of Cardiology, Medical University of Graz, Graz, Austria

## Abstract

**Background:**

To assess heart failure therapies in diabetic patients with preserved as compared to impaired systolic ventricular function.

**Methods:**

3304 patients with heart failure from 9 different studies were included (mean age 63 ± 14 years); out of these, 711 subjects had preserved left ventricular ejection fraction (≥ 50%) and 994 patients in the whole cohort suffered from diabetes.

**Results:**

The majority (>90%) of heart failure patients with reduced ejection fraction (SHF) and diabetes were treated with an ACE inhibitor (ACEi) or angiotensin receptor blocker (ARB) or with beta-blockers. By contrast, patients with diabetes and preserved ejection fraction (HFNEF) were less likely to receive these substance classes (p < 0.001) and had a worse blood pressure control (p < 0.001). In comparison to patients without diabetes, the probability to receive these therapies was increased in diabetic HFNEF patients (p < 0.001), but not in diabetic SHF patients. Aldosterone receptor blockers were given more often to diabetic patients with reduced ejection fraction (p < 0.001), and the presence and severity of diabetes decreased the probability to receive this substance class, irrespective of renal function.

**Conclusions:**

Diabetic patients with HFNEF received less heart failure medication and showed a poorer control of blood pressure as compared to diabetic patients with SHF. SHF patients with diabetes were less likely to receive aldosterone receptor blocker therapy, irrespective of renal function.

## Background

Heart failure is a major public health burden and the lifetime risk of developing heart failure in a 40 year old is around 20% [1]. About 50% of patients presenting with heart failure have normal ejection fraction (HFNEF) [2,3]. Recent research revealed that mortality of hospitalized patients with HFNEF is comparable to patients with systolic heart failure (SHF). However, in most heart failure trials, HFNEF patients were largely underrepresented.

Diabetes is a growing epidemiological burden and a major contributor to cardiovascular disease. In male patients with diabetes, the risk to develop heart failure is doubled in comparison to non-diabetic patients, but it is five times the risk of non-diabetic patients in women [4]. Moreover, diabetes is an independent predictor of poor outcome once SHF or HFNEF have developed [5,6]. Current treatment guidelines provide evidence for pharmacotherapy in diabetic patients with heart failure and adherence to guidelines is associated with improved outcome in both types of heart failure [7,8]. Of note, the fore-mentioned guideline does not specifically address patients with HFNEF [7].

The aim of the present study was to compare heart failure therapy in diabetic patients with SHF and HFNEF.

## Methods

### Patient cohorts

All subjects recruited within the German Heart Failure Network are characterized by an extensive standardizes baseline data set including information on socio-demographics, physical examination, heart failure aetiology and classification, cardiovascular risk factors, comorbidities, medication, ECG, echocardiography, coronary angiography, routine laboratory, and quality of life [9]. In all studies, a uniform baseline data set was obtained. All diagnostic procedures were performed in accordance with pre-specified Standard Operating Procedures. All individual studies were approved by local ethics committees.

For the current analysis, all patients from prospective follow-up studies with a diagnosis of heart failure were eligible.

In all patients echocardiography was performed according to guidelines of the American Society of Echocardiography (ASE) current at the time of data collection, including targeted M-Mode and Doppler techniques. All examinations were performed by physicians experienced in the technique and a pre-specified standard operation procedure regarding echocardiography was given. Preferable, the left ventricular ejection fraction was determined using the Simpson's model of discs. If patient's constitution did not allow sufficient appliance of the Simpson's method, visual estimation of LVEF was permitted too. Patients were classified as having SHF or HFNEF by echocardiographically determined left ventricular ejection fraction using a cut-off of 50%. Glomerular filtration rate was calculated by MDRD formula [10].

### Statistics

Data are presented as mean+/-SD or percentages. Estimates of percent of patients receiving a certain substance class are provided with 95% confidence intervals (CI).

Data were analysed by analysis of variance (quantitative) and logistic regression (frequencies), both including interaction terms for diabetes and left ventricular function. A two-tailed p < 0.05 was considered statistical significant. SPSS 15 (SPSS Inc., Chicago, IL) was used for analysis.

All individual studies were approved by local ethics committees. The authors had full access to and take full responsibility for the integrity of the data.

## Results

### Patient characteristics

3304 patients with heart failure from nine different sub-studies were included into this analysis. In the total sample, 711 patients (22%, 353 women) had preserved ejection fraction and 2593 patients (78%, 653 women) had SHF. 2310 patients (70%) were free of diabetes, 622 (19%) had mild diabetes (treated by diet or oral anti-hyperglycemic drugs) and 372 (11%) had severe diabetes (insulin-dependent treatment). Baseline characteristics of the study cohort are also displayed in table 1, showing significant differences for most variables according to presence of diabetes or SHF. Except for the SF-36 score, no significant interaction of effects of diabetes and ejection fraction was seen on all baseline variables.

**Table 1:** Baseline characteristics

	No Diabetes (n = 2310)		Diabetes (n = 994)		P value for respective effect	
					
					DM	EF
	**EF <50% (n = 1769)**	**EF ≥ 50% (n = 541)**	**EF <50% (n = 824)**	**EF ≥ 50% (n = 170)**		

Female (%)	23.5	49.2	28.9	51.2	0.005	<0.001

Age (years)	61.1 ± 14.3	63.1 ± 14.0	67.2 ± 10.3	69.4 ± 7.6	<0.001	<0.001

Body mass index (kg/m^2^)	26.9 ± 4.5	28.5 ± 5.1	29.1 ± 5.5	30.3 ± 5.3	<0.001	<0.001

Heart rate (bpm)	73.0 ± 13.5	69.7 ± 12.7	74.2 ± 12.5	72.4 ± 14.4	0.004	<0.001

Systolic blood pressure (mmHg)	121 ± 19	138 ± 23	125 ± 19	140 ± 21	<0.001	<0.001

Diastolic blood pressure (mmHg)	73 ± 11	79 ± 12	73 ± 11	77 ± 13	0.076	<0.001

Glomerular filtration rate (mL/min)	72 ± 24	75 ± 22	64 ± 26	68 ± 24	<0.001	0.002

Hb (mmol/mL)	8.7 ± 1.1	8.7 ± 1.0	8.4 ± 1.2	8.3 ± 1.1	<0.001	0.644

SF-36 score	52 ± 28	59 ± 28	44 ± 28	44 ± 27	<0.001	0.020*

* Significant interaction (p = 0.015) between diabetes and ejection fraction

• DM: Diabetes mellitus, EF: ejection fraction

### Treatment

**Figure 1:**
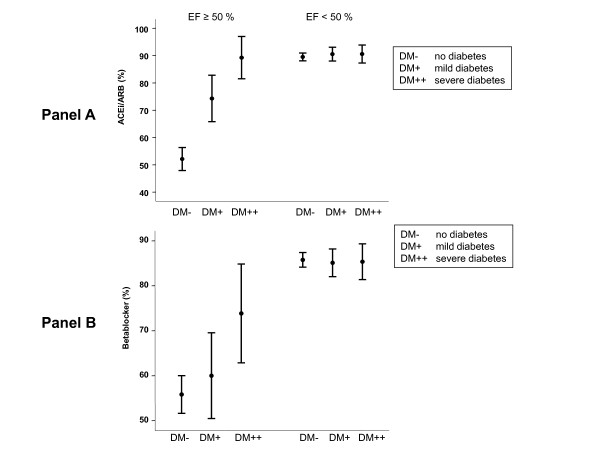
**Percentage of patients without diabetes (DM-), with mild diabetes (DM+, on diet or taking oral anti-hyperglycaemic drugs) and with severe diabetes (DM++, insulin-dependent diabetes) receiving different drugs (mean, 95% confidence interval)**. Panel A shows prevalence of treatment with ACE inhibitors or angiotensin receptor blockers, panel B shows therapy with beta-blockers. Data are separated by ejection fraction: Left side preserved ejection fraction (EF ≥ 50%), right side reduced ejection fraction (EF <50%).

**Figure 2:**
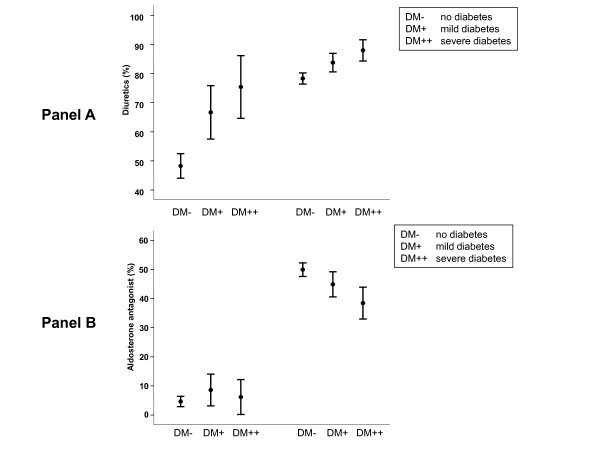
**Percentage of patients without diabetes (DM-), with mild diabetes (DM+, on diet or taking oral anti-hyperglycaemic drugs) and with severe diabetes (DM++, insulin-dependent diabetes) receiving different drugs (mean, 95% confidence interval)**. Panel A shows prevalence of treatment with diuretics, panel B shows therapy with aldosterone receptor blockers. Data are separated by ejection fraction: Left side preserved ejection fraction (EF ≥ 50%), right side reduced ejection fraction (EF <50%).

Overall, a high percentage of patients received the class I recommended therapy (i.e., angiotensin converting enzyme inhibitors [ACEi] or angiotensin-2 receptor-1 blocker [ARB] and beta-blockers). However, differences were observed between HFNEF and SHF patients. Blood pressure control in HFNEF was poorer than in SHF, and was poorer in patients with diabetes than in patients without diabetes (see table 1). There were also differences how guideline-recommended substance classes were applied in SHF and HFNEF for three important sub-groups: Patients free of diabetes, patients with mild diabetes and patients with severe diabetes [7]. As displayed in figure 1 around 90% of patients with SHF received ACEi or ARB (panel A) and beta-blockers (panel B) and neither the presence nor the severity affected the treatment with these substance classes (p = 0.409 and p = 0.724, respectively). By contrast, the intake of diuretics (figure 2, panel A) increased with the presence and severity of diabetes (p < 0.001, respectively) and a reduction in aldosterone receptor blocker usage with diabetes (Figure 2, panel B) was observed (p < 0.001).

As shown in figure 1, in HFNEF fewer patients received ACEi/ARB therapy than in SHF (p < 0.001). However, in contrast to SHF, both presence and the severity of diabetes were associated with a higher intake frequency of these substance classes (p < 0.001). A similar pattern was seen for beta-blockers (figure 1): The intake in HFNEF was reduced, but increased with the presence and severity of diabetes (p = 0.014). The presence of CAD was associated with a higher intake frequency of beta-blockers in HFNEF, but not in SHF. Comparable to SHF, diuretic therapy (figure 2) increased with the presence and severity of diabetes (p < 0.001). As also shown in figure 2, aldosterone receptor blockers were administered with lower frequency in HFNEF (p < 0.001); this association was unaffected by the presence and severity of diabetes (p = 0.198).

### Role of comorbidities

**Figure 3:**
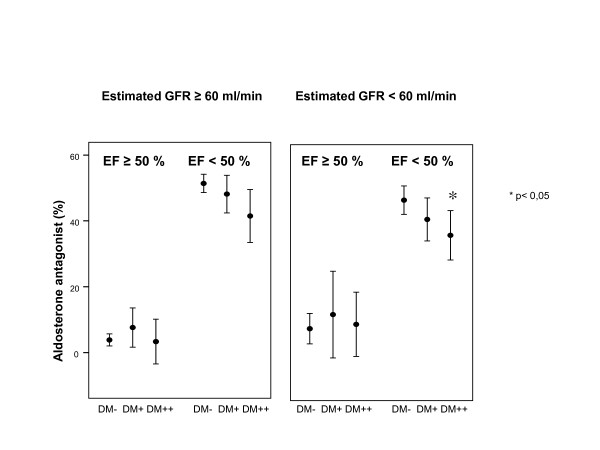
**Percentage of patients without diabetes (DM-), with mild diabetes (DM+) and with severe diabetes (DM++) receiving aldosterone receptor blockade therapy**. On the left panel, results are shown for patients with preserved renal function (GFR >60 ml/min), and on the right panel for patients with reduced renal function.

We investigated in detail the associations between renal dysfunction and the intake frequency of aldosterone receptor blockers, since hyperkalemia due to spironolactone poses a relevant risk in heart failure therapy [11]. Glomerular filtration rate was lower in SHF than in HFNEF and lower in diabetic than in non-diabetic patients and was lower in diabetic SHF than in diabetics HFNEF patients (see table 1). Figure 3 shows the treatment frequency of aldosterone receptor blockers stratified by preserved or reduced renal function and type of heart failure. In SHF, the percentage of patients receiving aldosterone receptor blockers decreased with the presence and severity of diabetes. Interestingly, this tendency was similar in patients with reduced and preserved renal function. Moreover, serum potassium levels were not different in patients without and with diabetes (p = 0.756 for HFNEF, p = 0.162 for SHF).

In the whole sample, the intake of ACEi/ARB was independently associated with the presence of diabetes, hypertension and renal dysfunction. In contrast, the intake of beta-blockers was associated with the presence of coronary artery disease and hypertension.

## Discussion

This is the first study to evaluate the most recent ESC/EASD recommendations for heart failure therapy in a large contemporary cohort of patients with heart failure. Moreover, no previous paper has studied a comparably high number of diabetic patients with heart failure and normal ejection fraction (HFNEF).

Our study has three major findings:

(1) Around 90% of diabetic patients with SHF are treated with ACEi/ARB and beta-blockers. This therapy pattern is not different from patients without diabetes.

(2) In contrast, diabetic patients with HFNEF are less likely to receive ACEi/ARB and beta-blockers. However, as compared to non-diabetic patients, they are more likely to receive these therapies. Blood pressure control in diabetic HFNEF patients is poorer than in diabetic SHF patients.

(3) Irrespective of renal function, diabetic SHF patients are less likely to receive aldosterone antagonists.

### Heart failure therapy in surveys

The percentage of patients with SHF receiving ACEi/ARB and betablockers was close to 90% and, hence, much higher than reported in previous surveys (e. g., the EuroHeart Survey) [12]. The large proportion of university centres and the exclusive recruitment in cardiology clinics may be an explanation [13]. An additional possibility is that slowly but steadily we reach a better implementation of guidelines in Germany.

### Heart failure therapy in patients with diabetes

There are very few, if any, clinical trials on heart failure treatment available that specifically address heart failure with the comorbidity diabetes. Therefore, recent recommendations carry a low level of evidence (level C) and are derived from subgroup analyses of patients suffering from diabetes in large heart failure trials [7]. In patients with SHF, the use of ACEi/ARB, beta-blockers and aldosterone antagonists have been shown to reduce morbidity and mortality (for review, see [1]). Aldosterone antagonists reduce mortality in heart failure patients with reduced ejection fraction and are of benefit in patients with systolic heart failure and recent myocardial infarction, with or without concomitant diabetes [14-16].

For HFNEF, there is yet no evidence-based drug-specific mortality reducing therapy available. Three trials investigated the use of ACEi and ARBs in HFNEF patients and failed to show a reduction in mortality [17-19]. Other treatment strategies, e.g. aldosterone receptor blockade, are currently investigated in clinical trials. Tight blood pressure control is the only recommendation supported by evidence in HFNEF, thus one would expect that this only recommendation would be strictly followed. However, our study shows that blood pressure control in HFNEF was inferior to SHF, the difference of about 15 mmHg in systolic blood pressure is partly explained by less pharmacotherapy.

The lack of evidence for heart failure treatment in HFNEF might explain the lower use of ACEi/ARBs, beta-blockers and aldosterone receptor blockers in patients with HFNEF. In contrast, data from the CHARM trial which was the largest trial to include patients with both SHF and HFNEF, showed that the presence of diabetes was of greater harm in HFNEF than in SHF patients [20]. It is unknown whether a more aggressive antihypertensive therapy develops benefit in patients with HFNEF, but it has been shown that diastolic dysfunction, a relevant pathophysiology in HFNEF, is improved by lowering blood pressure [21]. We were recently able to show that diastolic dysfunction is impaired along the whole diabetic continuum [22]. Furthermore, diastolic dysfunction, which is believed to be the responsible mechanism for the development of heart failure in the majority of patients with HFNEF, is known to be found more frequent in diabetic patients [23]. This increase in frequency of diastolic dysfunction in diabetes mellitus is thereby independent of renal function and can also be found under effective glycaemic control [24,25]. Given the proposed association of diabetes mellitus and blood pressure control with diastolic dysfunction, we argue for an improvement of antihypertensive therapy and of glycaemic control in patients with HFNEF. However, the hypothesis that strict blood pressure and glycaemic control is beneficial in diabetic HFNEF patients should be tested in a prospective randomised trial.

### Role of comorbidities

The use of ACEi/ARB or spironolactone may cause hyperkalemia, especially in patients with impaired renal function. A large population-based study from Ontario showed an increased incidence of hyperkalemia and subsequent mortality after the publication of the RALES trial and the rate of hyperkalemia in real-world is thought to be much higher than in clinical trials for various reasons (e. g. less frequent assessment of electrolytes and renal function, higher dosage of medication) [11,26,27]. In heart failure patients, diabetes has been shown to be an independent risk factor for the development of hyperkalemia and severe hyperkalemia (associated with hospitalization or death) [28,29]. Even with impaired renal function, however, heart failure patients with diabetes will benefit from ARB therapy and aldosterone receptor blockade, similarly to patients without diabetes [28,29]. Our data from a clinical practice setting suggest that the presence of the heart failure comorbidity diabetes restrains doctors from prescribing evidence-based therapy in heart failure patients - possibly in fear of side-effects, although these side-effects can be easily monitored by simple blood tests. As a consequence, we suggest that diabetic heart failure patients should receive appropriate doses of ACEi/ARB and/or aldosterone receptor blockers under a tight control of potassium and renal function markers, rather than withholding these potentially life-saving drugs. Moreover, future heart failure trials should focus on common comorbidities in heart failure populations (diabetes, impaired renal function, high age).

## Limitations

This is a cross-sectional observational study and although we included a high number of diabetic patients with HFNEF, the majority of included diabetic patients had SHF. In some of the studies, only patients with SHF were included. Thus, a stricter guideline-adherence for SHF in these SHF only studies may be an alternative explanation for the observed differences to HFNEF therapy. LVEF was not measured using a uniform methodology and intra- and inter-observer variability was not investigated and compared between the several medical centres. Although in most of all included patients LVEF was calculated according to Simpson's method, this may have biased our results.

Although we controlled for estimated glomerular filtration rate, we cannot rule out that more patients with diabetes had a history of acute renal failure and this could partly explain the lower frequency of aldosterone receptor intake in these patients.

## Conclusions

Despite significant improvements in the pharmacotherapy of heart failure, there is still a considerable undertreatment in diabetic heart failure patients with HFNEF. Diabetic SHF patients are less likely to receive aldosterone receptor blockade, irrespective of renal function and potassium levels.

## Competing interests

The authors declare that they have no competing interests.

## Authors' contributions

FE and RW participated in the design and the coordination of the study. They also participated in the acquisition of data throughout the study, in the analyses and interpretation of the results and drafted the manuscript. H-DD, SS, AR, RS, TN, CL, CEA and FM participated in the design of the study as well as in the acquisition of data throughout the study and were integrated in the analyses and interpretation of the results. GG participated in the design of the study and all aspects related to biometry. He has the full responsibility of the integrity of the data and the results. BP participated in the design and coordination of the study and helped to draft the manuscript. All authors critically read, revised and approved the final manuscript.
